# Value of 18-F-FDG PET/CT and CT in the Diagnosis of Indeterminate Adrenal Masses

**DOI:** 10.1155/2015/213875

**Published:** 2015-02-02

**Authors:** Nathalie Launay, Stéphane Silvera, Florence Tenenbaum, Lionel Groussin, Frédérique Tissier, Etienne Audureau, Olivier Vignaux, Bertrand Dousset, Xavier Bertagna, Paul Legmann

**Affiliations:** ^1^Department of Radiology, Cochin University Hospital, 27 rue du Faubourg St. Jacques, 75014 Paris, France; ^2^Sorbonne Paris Cité, Université Paris Descartes, 12 rue de l'École de Medicine, 75006 Paris, France; ^3^Department of Nuclear Medicine, Cochin University Hospital, 27 rue du Faubourg St. Jacques, 75014 Paris, France; ^4^Department of Endocrinology, Cochin University Hospital, 27 rue du Faubourg St. Jacques, 75014 Paris, France; ^5^Department of Anatomopathology, Cochin University Hospital, 27 rue du Faubourg St. Jacques, 75014 Paris, France; ^6^Department of Epidemiology and Biostatistics, Hôtel Dieu University Hospital, 1 Parvis Notre Dame-place Jean Paul II, 75004 Paris, France; ^7^Department of Digestive and Endocrine Surgery, Cochin University Hospital, 27 rue du Faubourg St. Jacques, 75014 Paris, France

## Abstract

The purpose of this paper was to study the value of 18-FDG PET/CT and reassess the value of CT for the characterization of indeterminate adrenal masses. 66 patients with 67 indeterminate adrenal masses were included in our study. CT/MRI images and 18F-FDG PET/CT data were evaluated blindly for tumor morphology, enhancement features, apparent diffusion coefficient values, maximum standardized uptake values, and adrenal-to-liver maxSUV ratio. The study population comprised pathologically confirmed 16 adenomas, 19 metastases, and 32 adrenocortical carcinomas. Macroscopic fat was observed in 62.5% of the atypical adenomas at CT but not in malignant masses. On 18F-FDG PET/CT, SUVmax and adrenal-to-liver maxSUV ratio were significantly lower in adenomas than in malignant tumors. An SUVmax value of less than 3.7 or an adrenal-to-liver maxSUV ratio of less than 1.29 is highly predictive of benignity.

## 1. Introduction

With the proliferation of cross-sectional imaging, detection of an incidental adrenal mass has become a common problem. Adrenal incidentalomas are detected on approximately 5 to 8% of all high-resolution abdominal imaging studies [1]. The majority of adrenal incidentalomas are adenomas. The diagnostic strategy is well established for adrenal adenomas and relies on the detection of intracellular lipids (using noncontrast CT or chemical shift MRI) and on the measurement of contrast washout kinetics on multiphasic CT [2–7]. However, about 12% of adrenal incidentalomas [8], including benign tumors, cannot be characterized by CT or MRI: these indeterminate adrenal masses are considered as suspect and may have CT follow-up and PET-CT exploration, and some may be surgically removed or biopsied in oncology patients. Being able to identify those indeterminate adrenal masses would avoid unnecessary follow-up, surgery, and making erroneous staging in oncology patients. The objective of our research is to study the value of 18-FDG PET/CT and reassess the value of CT for the characterization of indeterminate adrenal masses.

## 2. Materials and Methods

### 2.1. Patients

**Figure 1:**
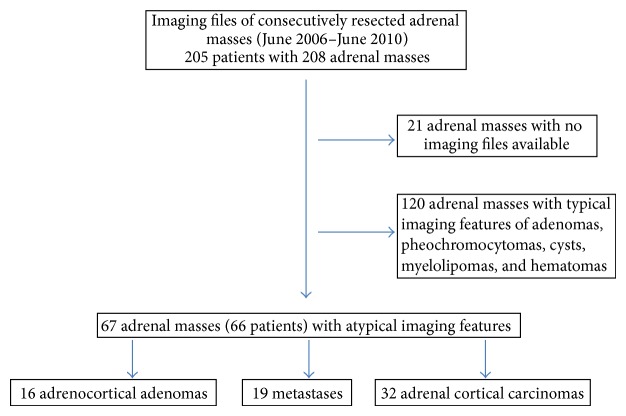
Flowchart of study enrollment.

Imaging files of 205 patients with 208 adrenal masses consecutively surgically resected or biopsied (2 metastases) between June 2006 and June 2010 were retrospectively reviewed. All patients had undergone thin-collimation computed tomography (CT) (unenhanced CT, contrast material-enhanced, with 10-minute delayed CT scan) or 18-F-FDG PET/CT. 19 patients (21 masses) for which CT or 18F-FDG PET/CT images were not available were excluded. Adrenal masses with typical imaging features of adenomas (92 masses with unenhanced density of less than 10 HU, an absolute percentage washout above 60%, or a signal intensity index above 20%), pheochromocytomas (23 masses), cysts (1 mass), and hematomas (4 masses) were excluded from the study. Finally 66 patients (67 masses) were included in our study. Our hospital includes a department of endocrinology, which is also a reference center for adrenal cortical carcinomas, contributing to their relatively large proportion (Figure 1).

Our institutional review board approved this retrospective study and waived the requirement for informed consent.

### 2.2. Imaging Techniques

Adrenal masses were examined by Siemens Sensation Scanner (16-detector) or Siemens Definition Scanner (64-detector). CT acquisition was performed before injection, at one minute after intravenous injection of 100 mL of nonionic contrast material with an iodine concentration of 300 mg/mL and at a delayed phase fixed at 10 min. 15-minute delayed protocol is classically used for washout analysis; however several studies have shown the absolute contrast enhanced percentage washout test accuracy using 10-minute delayed contrast enhanced CT [9, 10].

Imaging with 18-F-FDG-PET/CT was performed on a Gemini Dual Philips medical system (between 2006 and 2008) and on a Gemini TF 16 Philips medical system (between 2008 and 2010) that combines a helical dual slice CT and a PET machine, with an emission scan of 3 min duration per bed position. Patients fasted 12 h. Diabetic patients were prepared with oral antidiabetic medications or insulin the days before 18-F-FDG-PET/CT to obtain a glycemia less than 150 mg/dL. They were premedicated with diazepam and rested for 1 h. Imaging was performed 60 min after IV administration of 18-F-FDG (5 MBq/kg).

MR images were obtained using Siemens AVENTO MRI 1.5 T (Erlangen, Germany) or GE Signa MRI 1.5 T (Milwaukee, WI) closed MR system.

A phased-array body multicoil was used in all MR sequences (including T1, T2, diffusion, and in and out of phase acquisitions).

### 2.3. Image Analysis

All CT of 67 adrenal masses were retrospectively reviewed independently by two senior radiologists with more than ten years' experience in abdominal imaging (PL, SS), who were blinded to the pathologic diagnosis, and a junior radiologist with more than three years' experience (NL). Final interpretation was made by consensus. CT images were evaluated for their morphologic features and their contrast enhancement patterns. CT scans were evaluated for their morphologic and enhancement features with the soft-tissue window setting. To assess the morphologic features of the masses, the observers measured the maximal diameter of the tumors, attempted to determine the contour, the homogeneity of the lesions on unenhanced images, the presence of macroscopic fat, calcifications, hemorrhage, solid tissue nodules, walls, and cystic/necrotic regions. The textures of the lesions were classified as homogeneous or heterogeneous on unenhanced CT images. For analysis of tumor enhancement features, the observers determined the following: homogeneous or heterogeneous enhancement patterns and the relative and absolute washout values. For the CT images, an absolute percentage washout (APW) was calculated as follows from the attenuation values recorded on the unenhanced, dynamic, and delayed images absolute: APW = (enhanced − delayed)/(enhanced − unenhanced) × 100%. Concerning 18-F-FDG PET/CT data, SUVmax and adrenal to liver maxSUV ratio were considered for statistical analysis, as previously described by Groussin et al. who proposed an adrenal to liver maxSUV ratio cutoff value of 1.45 to distinguish between adrenocortical carcinomas and adrenal adenomas, with a sensitivity of 100% and a specificity of 88%, which was higher than the specificity of 70% obtained with the SUVmax cutoff value [11].

### 2.4. Statistical Analysis

To determine the differences in imaging features between atypical adenomas, metastases, and adrenocortical carcinomas, the Student test, the Mann-Whitney test, the *χ*²-test, and the Fisher exact test were used. Statistical analysis was performed using Stata Logical. For each analysis, a *P* value of less than 0.05 was considered to indicate a significant difference. The discriminative properties of 18F-FDG PET/CT were investigated by receiver-operating characteristic (ROC) analysis. The area under the curve was assessed, and the sensitivity and specificity were determined for an optimal cutoff of the SUVmax and of the adrenal to liver maxSUV ratio.

## 3. Results

The mean age of the sixty-seven patients was 56.5 years ± 4. Thirty-three men and thirty-four women were included in the study. Sixteen adenomas, eighteen metastases, and thirty-one adrenocortical carcinomas were examined by CT. Twelve adenomas, eight metastases, and twenty-three adrenocortical carcinomas were examined by 18F-FDG PET/CT.

### 3.1. Adrenocortical Adenomas: Clinical, Imaging, and Pathological Features

The mean age of the patients with adrenocortical adenomas was 64 years ± 4 (nine women (mean age, 64.1 ± 8.4; age range, 55.7–72.5 years)) (seven men (mean age, 63.9 ± 3; age range, 61–67 years)). 87.5% of the atypical adenomas were nonsecreting adenomas and 12.5% were Cushing's adenomas (urinary free cortisol > 100 mcg/24 h and adrenocorticotropic hormone (ACTH) < 6 pg/mL).

**Figure 2:**
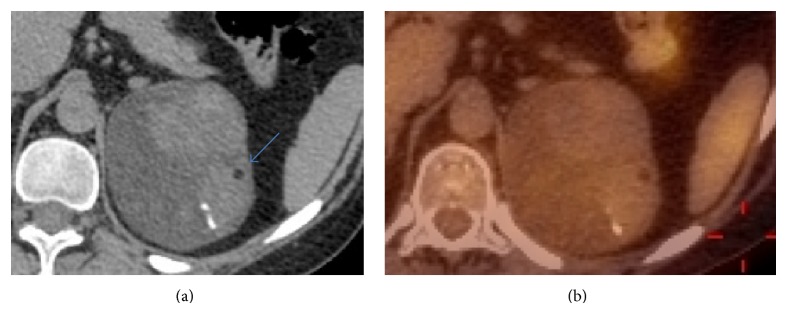
42-year-old woman with Cushing syndrome presenting a left heterogeneous adrenal mass, with a cystic area containing walls, calcifications, macroscopic fat (blue arrow), and hemorrhage. (a) Unenhanced CT. (b) 18-F-FDG PET/CT (SUVmax: 2). Histologic diagnosis: proliferation of adrenal cortical cells in well vascularized interstitial tissue. No sign of malignancy.

**Figure 3:**
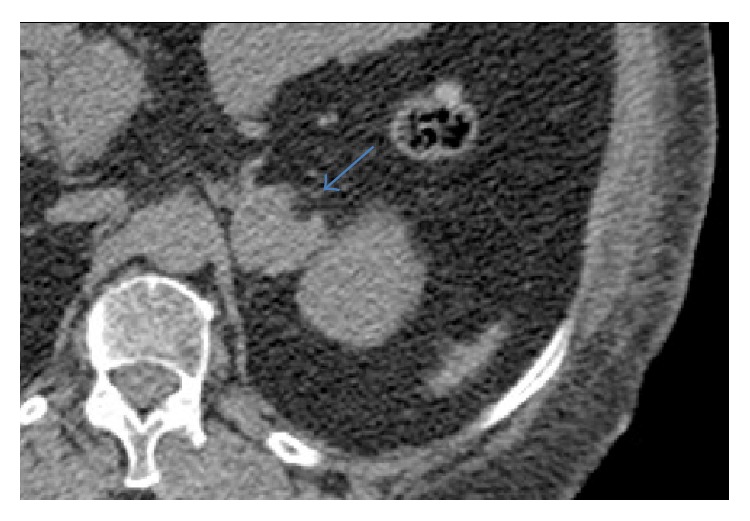
67-year-old woman with Cushing syndrome presenting a left heterogeneous adrenal mass, showing calcifications, and macroscopic fat (blue arrow) on an unenhanced CT. Histologic diagnosis: adrenal cortical adenoma.

Sixteen adrenocortical adenomas were examined by CT: their margins were regular and well-defined in all cases; 93.75% of them were heterogeneous presenting macroscopic fat (62.5%) (evaluated with CT sequences with an unenhanced density lower than −40 HU) (Figures 2 and 3), 31.3% of them contained cystic areas, 12.5% of them contained hemorrhagic areas and 81.3% of them contained calcifications.

None of them presented solid tissue nodules and 18.8% of them contained walls. Their mean unenhanced density was 27 HU ± 6.7 (range, 14–37 HU). Eleven adenomas were examined by multiphase CT: 36.3% of them presented a washout, with a mean absolute value of 30.5% (range, 17–51%). The other adenomas (63%) showed persistent enhancement throughout the 10 min delayed phase (zero washout). Twelve adenomas were examined by 18F-FDG PET/CT. Their mean SUVmax was 3.24 (range, 1.69–4.79) and their mean (SUVmax)/(SUV liver) value was 1.33 (range, 0.57–2.09). 18F-FDG PET/CT images did not show significant uptake (>2 times liver uptake value) for 11 adenomas. However one adenoma showed a high uptake value (SUVmax: 10.3, adrenal to liver maxSUV ratio: 5); this adenoma at pathological analysis had a Weiss score of 2. Adrenal tumors with a Weiss score of between 0 and 2 are considered to be benign, while adrenal tumors with a Weiss score of more than 3 are considered to be malignant [12]. At pathologic examinations, thirteen adrenal adenomas (81.2%) presented a Weiss score of 0, two (12.5%) presented a Weiss score of 1, and one (6.2%) presented a Weiss score of 2. Six adenomas (37.5%) contained focal regions of hemorrhage, and three (18.7%) contained macroscopic fat. Histologically, none of them contained areas of necrosis.

### 3.2. Adrenal Metastases: Clinical, Anatomopathological, and Imaging Features

**Figure 4:**
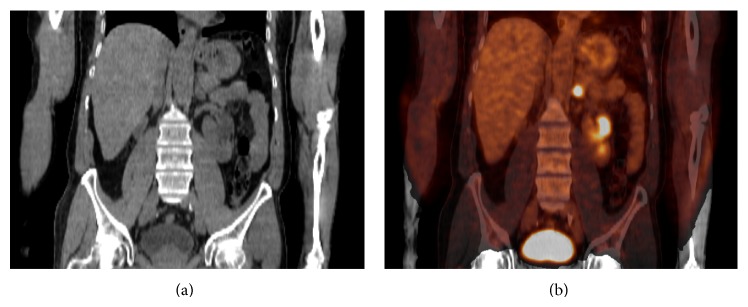
64-year-old patient with known melanoma. (a) Unenhanced CT showing left homogeneous adrenal mass, with poorly defined margins. (b) 18-F-FDG PET/CT showing intensive FDG uptake of the left adrenal mass with an SUVmax measured at 8.1. Histologic diagnosis: adrenal metastases of melanoma.

The female/male ratio was 0.3. Eighteen metastases were examined by CT. Mean unenhanced density was 34 HU. Six metastases were examined by multiphasic CT and their mean washout value was 11%. 66% of them presented zero washout. None of the metastases contained macroscopic fat or calcifications. 5% of them contained areas of hemorrhage. Eight metastases were examined by 18F-FDG PET/CT. Adrenal metastases showed high uptake value (Figure 4) with a mean SUVmax of 7.56 (range, 4.9–10.2) and an adrenal to liver maxSUV ratio of 2.68 (range, 1.7–3.6). At pathologic examinations, eight metastases contained area of necrosis and three metastases contained hemorrhage.

### 3.3. Adrenocortical Carcinomas: Clinical, Anatomopathological, and Imaging Features

**Figure 5:**
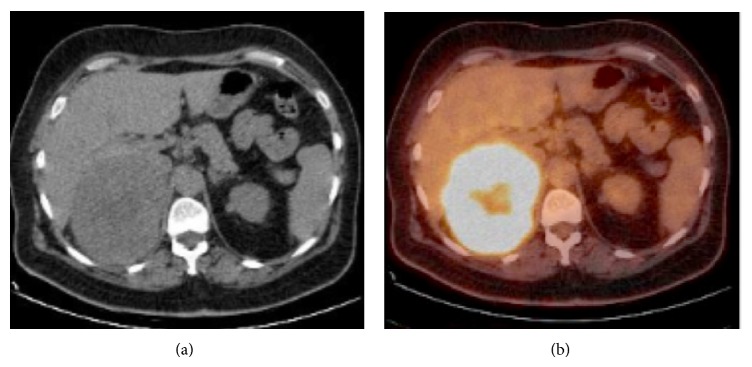
61-year-old woman with symptoms of androgen excess. (a) Unenhanced CT showing right heterogeneous large adrenal mass. (b) 18-F-FDG PET/CT showing intensive FDG uptake of the right adrenal mass with an SUVmax measured at 14.4. Histologic diagnosis: adrenal cortical carcinoma with a Weiss score of 7.

Mean age was 49.6 years ± 6.5 for adrenal cortical carcinomas and female/male ratio was 1.9. 56% of the adrenocortical carcinomas with available biological data (26 masses) were nonsecreting. 18.5% secreted both androgen and steroid hormones, 15% caused Cushing syndrome, 7.4% secreted only androgen, and 3.7% secreted estrogen. Thirty-one adrenocortical carcinomas were examined by CT. The mean of maximum diameters was 83 mm ± 12 mm. Only 41.2% of the adrenal cortical carcinomas had well defined margins. None of the adrenal cortical carcinomas presented macroscopic fat. 58.8% of the adrenal cortical carcinomas presented ill-defined heterogeneous cystic areas (Figure 5). Twenty-three adrenocortical carcinomas were examined by 18F-FDG PET/CT. Mean SUVmax was 11.38 (range, 8.61–14.15) and mean adrenal to liver maxSUV ratio was 4.3 (range, 3.31–5.28) for adrenal cortical carcinomas (Figure 5). Pathologically, 73% of these masses contained areas of necrosis.

### 3.4. Adrenocortical Adenomas versus Metastases

The female/male ratio was 0.3 for metastases and 1.28 for adrenocortical adenomas.

The mean unenhanced density was 27 HU for adrenocortical adenomas and 34 HU for metastasis on CT (*P* = 0.016) (Table 1).

Margins were significantly better defined for adenomas than for metastases. 62.5% of adrenal adenomas presented macroscopic fat, while none of the metastases contained macroscopic fat (Table 1). 81.3% of the adrenocortical adenomas were calcified, while none of the metastases contained calcifications. 12.5% of the adrenocortical adenomas and 6% of the adrenal metastasis contained hemorrhagic deposits (*P* > 0.05). There was no significant signal difference in diffusion sequences. On 18F-FDG PET/CT, maximum standardized uptake values (SUVmax) were significantly lower for adenomas (3.24) than for metastases (7.56) (*P* < 0.05). Adrenal to liver maxSUV ratio was significantly lower for adenomas (1.33) than for metastases (2.68) (Table 1).

### 3.5. Adrenocortical Adenomas versus Adrenocortical Carcinomas

Mean age was 64 years ± 4 for adrenocortical adenomas and 49.6 years ± 6.5 for adrenal cortical carcinomas (<0.05). Female/male ratio was 1.28 for adrenocortical adenomas and 1.9 for adrenocortical carcinomas. The mean of maximum diameters was 4.8 cm ± 1.4 for adrenocortical adenomas and 8.3 cm ± 1.2 for adrenal cortical carcinomas (*P* = 0.001) (Table 1). All adenomas had well-defined margins, while only 41.2% of adrenal cortical carcinomas did. 62.5% of the adrenal adenomas presented macroscopic fat on CT, while none of the adrenal cortical carcinomas did. 58.8% of the adrenal cortical carcinomas presented ill-defined heterogeneous cystic areas. On 18F-FDG PET/CT, maximum standardized uptake values (SUVmax) were significantly lower for adrenal adenomas (3.24) than for adrenal cortical carcinomas (11.1) (*P* < 0.05). Adrenal to liver maxSUV ratio was significantly lower for adenomas (1.33) than for adrenocortical carcinomas (4.3, *P* < 0.05). Histologically, 73% of these masses contained areas of necrosis. 81.3% of the adrenocortical adenomas and 20.6% of the adrenal cortical carcinomas were calcified (*P* < 0.001).

### 3.6. Adrenocortical Adenomas versus Malignant Tumors: Main Results

**Figure 6:**
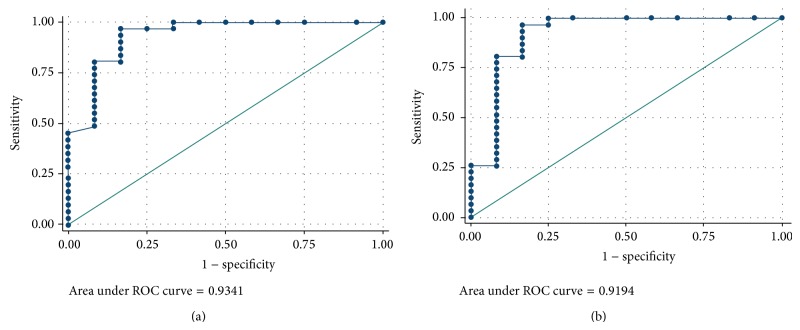
ROC curves (vertical axis: sensitivity; horizontal axis: 1−specificity) generated from SUVmax and adrenal to liver maxSUV ratio. (a) ROC curve generated from SUVmax. (b) ROC curve generated from adrenal to liver maxSUV ratio.

62.5% of adrenal adenomas presented macroscopic fat on CT, while none of the malignant masses did (*P* < 0.05). Presence of macroscopic fat tended to indicate benignity. Calcifications, hemorrhagic areas, heterogeneity, and the presence of walls had no diagnostic value for or against malignancy. On 18F-FDG PET/CT, maximum standardized uptake values (SUVmax) were significantly lower in the adenomas (3.24) than in malignant tumors (10.3) (*P* < 0.05). Adrenal to liver maxSUV ratio was significantly lower in atypical adenomas (1.33) than in malignant tumors (3.9, *P* < 0.05).  Figure 6(a) displays the ROC plots for maxSUV: discrimination was very good with an area under ROC curve of 0.93 (95% confidence interval (CI) 0.84–1.00). Using 3.7 as a cutoff value for SUVmax, a sensitivity of 96.7% (95% CI 0.83–0.99) and a specificity of 83.3% (95% CI 0.55–0.95) were achieved to distinguish between adrenal adenomas and adrenal malignant masses.  Figure 6(b) displays the ROC plots for adrenal to liver maxSUV ratio: discrimination was good with an area under ROC curve of 0.91 (95% confidence interval (CI) 0.79–1.00). Using 1.29 as a cutoff value for adrenal to liver maxSUV ratio, a sensitivity of 96.7% (95% CI 0.83–0.99) and a specificity of 83.3% (95% CI 0.55–0.95) were achieved to distinguish between adrenal adenomas and adrenal malignant masses.

## 4. Discussion

Although progress has been made at imaging with CT and MRI for the diagnosis of adrenal masses, some incidentalomas remain undetermined. Though the diagnostic strategy is well established for typical adrenal adenomas, pheochromocytomas, cysts, myelolipomas, or hematomas, a significant number of adrenal masses remain indeterminate. These tumors are usually removed, with potential surgical complications, such as splenectomy, kidney removal, or massive hemorrhage. Among these tumors, adenomas with atypical imaging features, which are benign, cannot be distinguished from adrenal malignant masses [13, 14]. The characterization of the atypical adenomas is crucial in the following 3 cases.

- Incidentalomas: the diagnosis of an adenoma would be followed up by CT examinations only, whereas further investigations and treatments would be necessary for metastases or adrenocortical carcinomas.
- Secreting masses: it is important to differentiate adenomas and adrenal cortical carcinomas in the case of Cushing syndrome. The prognosis and the treatment would be different depending on the nature of the tumor [15].
- Patients with known cancer: the prognosis and the treatment would completely change according to the tumor's nature (adenoma or metastases).

Until now, only one study focused on a series of large adrenal adenomas with histologically atypical features. 30 adenomas larger than 5 cm with histologically atypical features were compared to 24 adrenal cortical carcinomas. This study could not show any significant difference between the imaging features of atypical large adenomas and adrenal cortical carcinomas [15]. More recent studies focused on the evaluation of new techniques such as spectroscopy MRI, dynamic enhanced MRI, diffusion weighted MRI, perfusion CT, and contrast-enhanced ultrasonography for the characterization of adrenal masses [16–24]. A recent study focused on the value of spectroscopy MRI in order to distinguish adrenal adenomas, pheochromocytomas, metastases, and adrenal cortical carcinomas, with interesting results concerning choline/creatine and 4.0–4.3/creatine ratios. However, most of the 38 adenomas included presented typical imaging features, except one which could not be characterized by CT [24]. Another study examined the value of dynamic contrast enhanced MRI in the differential diagnosis of adrenal adenomas and malignant adrenal masses, with promising results concerning contrast enhancement patterns and time-to-peak values. However most of the 48 adenomas included were typical, except 4 adenomas which could not be characterized after chemical shift on MRI [22]. Other studies focused on the improvement of techniques already used to characterize adrenal masses, such as chemical shift sequences [25–27] or contrast washout kinetics on multiphase CT. In our study, macroscopic fat was only found in adenomas: 62.5% of adrenal adenomas presented macroscopic fat on CT, while none of the malignant masses did (*P* < 0.05). In the literature, however, 3 cases of adrenocortical carcinomas containing macroscopic fat were reported [28–30]. The reported cases presented similar imaging features including large size, heterogeneous peripheral enhancement, and a relative small amount of macroscopic fat. Though the presence of macroscopic fat in an adrenal tumor usually indicates benignity, adrenocortical carcinomas should be considered as differential diagnosis if features suggesting malignancy are associated. Calcifications, hemorrhagic areas, heterogeneity, and the presence of walls (except for metastasis) had no diagnostic value for or against malignancy. Our study results show that poorly defined margins tend to indicate malignancy (*P* < 0.05).

On 18F-FDG PET/CT, maximum standardized uptake values (SUVmax) were significantly lower in the adenomas (3.24) than in malignant tumors (10.3) (*P* < 0.05). Adrenal to liver maxSUV ratio was significantly lower in atypical adenomas (1.33) than in malignant tumors (3.9, *P* < 0.05). One benign atypical adenoma showed a high SUVmax; however it also presented the highest Weiss score, 2, among our series of adenoma. Using 3.7 as a cutoff value for SUVmax or 1.29 as a cutoff value for adrenal to liver maxSUV ratio, a sensitivity of 96.7% (95% CI 0.83–0.99) and a specificity of 83.3% (95% CI 0.55–0.95) were achieved to distinguish between adrenal adenomas and adrenal malignant masses. Boland et al. demonstrated that quantitative PET with the use of mean or maximal SUVs had a sensitivity of 97% and a specificity of 87% for characterizing adrenal masses as malignant [31]. Their meta-analysis showed that qualitative PET had the same sensitivity (97%) and a better specificity (91%) for characterizing adrenal masses as malignant. Our results also are similar to those demonstrated by Groussin et al. who demonstrated a high sensitivity and specificity of quantitative PET/CT for distinguishing between adrenal adenomas and adrenocortical carcinomas especially using liver maxSUV ratio with a sensitivity of 100% and a specificity of 88% for the cutoff value of 1.45 and a sensitivity of 100% and a specificity of 70% using SUVmax with a cutoff value of 3.4. 18F-FDG PET/CT allowed a correct diagnosis in 13 of 15 adenomas which remained undetermined at CT [11]. 18F-FDG PET/CT has a high sensitivity and specificity to characterize undetermined adrenal masses [31, 32] and the value of SUVmax may be correlated to the Weiss score. In oncology patients presence of macroscopic fat as well as low SUV uptake in undetermined adrenal masses at CT and MRI should be taken in consideration together with biopsy in order not to worsen prognosis. Same imaging features should be used to evaluate diagnosis of adrenocortical adenomas versus malignant masses. Presence of macroscopic fat in the case of well-defined adrenal incidentalomas of less than 5 cm would be a good indicator to follow up the mass instead of resecting it.

There were some limitations to our study. First it was limited by its retrospective nature. However, our study provides a relatively large series of pathologically proven adrenocortical atypical adenomas, adrenal metastases, and adrenocortical carcinomas, seen on CT, MRI, or 18-F-FDG PET/CT. Second, the study only focused on excised masses, because a pathologically proven diagnosis was necessary. Moreover, diagnostic problems generally occurred for operable patients without extensive metastatic diseases. Third, this study included a large portion of malignant masses. It may be assumed that in a different patient demographic with fewer adrenocortical carcinomas the sensitivity reported for the 3.7 SUV cutoff will decrease and the specificity will not be significantly impacted.

In conclusion, this large series of all pathologically confirmed adrenal masses including adrenocortical atypical adenomas, adrenal metastases, and adrenocortical carcinomas showed that the presence of macroscopic fat on CT is an important indicator of benignity for adrenal tumors that remain indeterminate. 18F-FDG PET/CT is highly sensitive and specific for distinguishing between benign and malignant adrenal tumors especially in case of indeterminate adrenal masses.

## Figures and Tables

**Table 1:** Imaging features of adrenocortical adenomas, adrenal metastases, and adrenocortical carcinomas.

Characteristic	Adrenocortical adenomas	Adrenal metastases	Adrenocortical carcinomas
Size (cm)	4.8 ± 1.4	4.1 ± 1.1	8.3 ± 1.2 (*P* = 0.001)
Well-defined margins (%)	100	15.8 (*P* = 0.000)	41.2 (*P* = 0.000)
Homogeneous (%)	6.25	72.2 (*P* = 0.001)	30.3
Hemorrhage (%)	12.5	6	12.5
Calcifications (%)	81.3	0 (*P* = 0.000)	20.6 (*P* = 0.000)
Cyst/necrosis (%)	31.3	29.4	58.8
Macroscopic fat (%)	62.5	0 (*P* = 0.000)	0 (*P* = 0.000)
Walls (%)	18.8	0 (*P* = 0.04)	22
SUVmax	3.24 ± 1.55	7.5 ± 2.7 (*P* = 0.003)	11.38 ± 2.77 (*P* = 0.000)
SUVmax/SUVliver	1.33 ± 0.76	2.68 ± 0.9 (*P* = 0.003)	4.3 ± 0.98 (*P* = 0.000)
ADC (mm^2^/s)	1842	1035	986 (*P* = 0.045)
Unenhanced density (HU)	27 ± 6	34 ± 6	33.6 ± 5
